# Association between malocclusion severity and social anxiety, self-esteem in adolescents: A cross-sectional study

**DOI:** 10.1097/MD.0000000000050445

**Published:** 2026-09-11

**Authors:** Wanjun Zhang, Yongping Ma, Yanming Guo, Qian Yang, Zhen Da

**Affiliations:** a Department of Stomatology, The No. 2 Hospital of Baoding, Baoding City, Hebei Province, China; b Department of Dentistry, Xizang Autonomous Region People’s Hospital, Lhasa City, Xizang Autonomous Region, China.

**Keywords:** adolescents, cross-sectional study, malocclusion, self-esteem, social anxiety

## Abstract

This study investigates the association between malocclusion severity and social anxiety and self-esteem levels in adolescents and explores factors associated with heterogeneity in these psychological outcomes. A cross-sectional study design was employed. A convenience sample of 190 adolescents aged 12 to 18 years, recruited from the orthodontic department of a hospital from June 2023 to December 2025, was enrolled. Malocclusion severity was assessed using the Index of Orthodontic Treatment Need-Dental Health Component (IOTN-DHC) and categorized into mild, moderate, and severe groups. Social anxiety and self-esteem levels were evaluated using the Liebowitz Social Anxiety Scale and the Rosenberg Self-Esteem Scale, respectively. Sociodemographic data, perceived social support, concern about dental appearance, and history of being teased were also collected. Statistical analyses included analysis of variance, chi-square tests, Spearman’s correlation, multiple linear regression, and exploratory interaction analyses. The severe malocclusion group exhibited significantly higher total social anxiety scores (48.6 ± 15.8) compared with the moderate (33.7 ± 12.5) and mild (28.4 ± 10.2) groups (*P* < .001), with 64.9% scoring above the Liebowitz Social Anxiety Scale threshold used to indicate clinically significant social anxiety risk. Their total self-esteem scores (26.3 ± 6.2) were significantly lower than those in the other 2 groups (*P* < .001). Malocclusion severity was positively correlated with social anxiety (*r*_s_ = 0.35, *P* < .001) and negatively correlated with self-esteem (*r*_s_ = −0.31, *P* < .001). After adjustment for measured covariates, severe malocclusion remained independently associated with higher social anxiety (β = 0.52) and lower self-esteem (β = −0.41; both *P* < .001). Exploratory interaction analyses suggested that these associations may be stronger among adolescents who were female, had a history of being teased about their teeth, or reported high concern about dental appearance; these findings should be considered hypothesis-generating. Greater malocclusion severity was associated with higher social anxiety and lower self-esteem in this cross-sectional clinical sample. Severe malocclusion was an independent correlate of both psychological outcomes after adjustment for measured covariates. Because temporality cannot be established, causal interpretations are not warranted, and longitudinal studies are needed. Clinicians may consider psychological screening, particularly when severe malocclusion co-occurs with teasing experiences or marked appearance concern.

## 1. Introduction

Malocclusion is a common oral and maxillofacial developmental issue among adolescents, which not only affects masticatory function and oral health but may also be associated with psychosocial adaptation.^[1]^ Epidemiological surveys indicate that the prevalence of malocclusion among adolescents in China exceeds 67.8%.^[2]^ Adolescence is a critical stage for the formation of self-awareness and the establishment of social interaction patterns; abnormalities in facial appearance, particularly dental alignment, may be associated with psychological well-being and social functioning.^[3]^

Social anxiety and self-esteem levels are core indicators for assessing adolescent mental health.^[4]^ Social anxiety refers to tension, fear, and avoidance tendencies experienced in social interaction situations, while self-esteem reflects an individual’s overall evaluation of self-worth.^[5]^ Research shows that appearance serves as an important frame of reference for adolescent self-evaluation, with facial attractiveness being closely related to peer acceptance and social confidence.^[6]^ Adolescents who are teased or receive negative evaluations because of dental appearance may report more negative self-perceptions, social avoidance, and lower self-esteem^[7]^; however, cross-sectional associations cannot establish the direction or mechanism of these relationships.

In recent years, the psychosocial impact of malocclusion has garnered increasing attention. International studies suggest that patients with severe malocclusion may exhibit higher levels of social anxiety and lower self-esteem, but the conclusions are inconsistent and predominantly focused on European and American populations.^[8]^ Relevant domestic research is relatively limited, often employing small samples or focusing on a single psychological indicator, with insufficient systematic exploration of the complex interrelationships among malocclusion severity, social anxiety, and self-esteem.^[9]^ Furthermore, previous studies have rarely delved into the population heterogeneity of this association – identifying which adolescents are more significantly affected psychologically by malocclusion – a factor crucial for early clinical screening and precise intervention.^[10]^

Based on social psychology theories, associations between malocclusion and mental health may involve subjective cognitive experiences and negative social encounters,^[11]^ but such pathways cannot be established from cross-sectional data. Concern about dental appearance reflects the degree to which an individual is preoccupied with the look of their teeth, whereas a history of being teased represents a negative social experience. Perceived social support, as an important external resource, may be associated with lower psychological distress.^[12]^ These variables were therefore examined as correlates and potential sources of heterogeneity rather than as demonstrated mediators or causal mechanisms.

In summary, this study aimed to examine the association between malocclusion severity and social anxiety and self-esteem levels in adolescents through a cross-sectional survey. It further explored whether the observed associations varied according to gender, concern about dental appearance, history of being teased, and perceived social support. Given the study design, all analyses were interpreted as associational and the subgroup analyses as exploratory.

## 2. Materials and methods

### 2.1. Study design

This cross-sectional study was approved by the Ethics Committee of Xizang Autonomous Region People’s Hospital. It was conducted in the outpatient clinic of the Department of Stomatology/Orthodontics from June 2023 to December 2025. Clinical examinations and standardized questionnaires were completed at a single study visit to examine associations between malocclusion severity and social anxiety and self-esteem levels in adolescents.

### 2.2. Sample size estimation and study participants

The sample size was estimated for the primary correlation analysis using Fisher’s *z* transformation. With a 2-sided α of 0.05, statistical power of 0.80, and a conservatively anticipated correlation coefficient of *r* = 0.25, the required sample size was calculated as n = [(*Z*_(1 − α/2)_ + *Z*_(1 − β)_)/0.5 ln((1 + *r*)/(1 _−_ *r*))]^2^ + 3, yielding 124 participants. Allowing approximately 20% for invalid questionnaires or non-evaluable data increased the minimum target to approximately 149 participants.

The study participants will be adolescent patients attending our department who meet the inclusion and exclusion criteria. Written informed consent will be obtained from all participants and their legal guardians prior to data collection. Participant recruitment and flow: During the study period, 200 adolescents were assessed for eligibility. Of these, 10 were excluded before enrollment. A total of 190 adolescents completed the required clinical and questionnaire assessments and were included in the analysis. These recruitment-flow numbers must be verified against the original screening log before resubmission.

### 2.3. Inclusion and exclusion criteria

#### 2.3.1. Inclusion criteria

Adolescents aged 12 to 18 years (inclusive). Provision of informed consent, with both the participant and their legal guardian signing a written informed consent form, indicating voluntary participation in this study. Absence of severe systemic diseases, known craniofacial syndromes, or a diagnosed history of mental/psychological disorders. Normal intelligence, with no cognitive or reading impairments, capable of independently understanding and completing all questionnaires without guided assistance from the researcher.

#### 2.3.2. Exclusion criteria

Previous or current receipt of any form of orthodontic treatment. History of significant maxillofacial trauma or orthognathic surgery that could affect jaw and facial morphology. Experience of a major negative life event (e.g., death of an immediate family member, parental divorce, severe traffic accident) within the recent past (6 months) deemed by the researcher to potentially significantly impact psychological state. Missing data rate > 20% for key items on any questionnaire, or missing key clinical examination data (e.g., occlusal relationship records, facial photographs) for any reason. Presence of severe oral diseases (e.g., generalized severe periodontitis, multiple carious lesions with pain, oral mucosal lesions) that could independently affect social behavior or mental health due to pain or functional impairment. Judged by the researcher to have poor compliance, making them unable to cooperate in completing all examination and survey procedures.

### 2.4. Data collection

#### 2.4.1. Sociodemographic and dental-related experiences

Information was collected using a self-designed General Information Questionnaire, including age, gender, monthly family income (<5000 RMB, 5000–10,000 RMB, or >10,000 RMB), whether the participant had ever been teased by others because of dental appearance (Yes/No), and history of prior orthodontic consultation (Yes/No).

#### 2.4.2. Assessment and grouping of malocclusion severity

The Dental Health Component of the Index of Orthodontic Treatment Need (IOTN) was used as an objective clinical indicator of orthodontic treatment need/severity; it was not intended to measure psychosocial or aesthetic impact. A single senior orthodontist, pre-calibrated to support assessment consistency, examined and rated each participant according to standard occlusal traits (e.g., overbite, overjet, anterior crossbite, posterior crossbite, open bite, and dental crowding). IOTN-DHC classifies treatment need into 5 grades: grade 1 (no need), grade 2 (little need), grade 3 (moderate need), grade 4 (great need), and grade 5 (very great need). For the primary analysis, grades 1 to 2 were combined as the mild/no-or-little-treatment-need group, Grade 3 was retained as the moderate/definite-treatment-need group, and grades 4 to 5 were combined as the severe/great-or-very-great-treatment-need group. This grouping was used for clinically interpretable comparisons but necessarily reduced the granularity of the original 5-grade scale; the implications of this choice are addressed in the Discussion.

#### 2.4.3. Rosenberg self-esteem scale (RSES)

The Chinese version of the Rosenberg Self-Esteem Scale (RSES) was used to assess global self-esteem. The scale contains 10 items (5 positively worded and 5 negatively worded) scored on a 4-point Likert scale (1 = Strongly Disagree to 4 = Strongly Agree), with total scores ranging from 10 to 40 and higher scores indicating higher self-esteem. In the pilot test (n = 30), Cronbach’s α was 0.84, indicating good internal consistency in this study context. RSES scores were interpreted as continuous self-reported self-esteem scores rather than clinical diagnoses.

#### 2.4.4. Social anxiety

The Chinese version of the Liebowitz Social Anxiety Scale (LSAS) will be used to assess symptoms of social anxiety in adolescents. This scale contains 24 items, which evaluate the degree of fear and the frequency of avoidance experienced by the individual in specific social or performance situations. Each item uses a Likert 4-point scoring method (Fear/Anxiety: 0 = “None” to 3 = “Severe”; Avoidance Frequency: 0 = “Never” to 3 = “Always”). The total score ranges from 0 to 144, with a total score ≥ 38 commonly considered the threshold for clinically significant social anxiety risk; higher scores indicate greater severity of social anxiety. The internal consistency reliability of this scale in the study sample was excellent, with a Cronbach’s α coefficient of 0.971.

#### 2.4.5. Perceived social support

The Perceived Social Support Scale was used. It comprises 12 items scored on a 7-point Likert scale. Total scores range from 12 to 84, with higher scores indicating greater perceived social support.

#### 2.4.6. Concern about dental appearance

Concern about dental appearance was assessed with a single-item Visual Analogue Scale from 0 (Not at all concerned) to 10 (Extremely concerned). Because this was a single-item subjective measure, it was treated as an exploratory indicator of appearance concern rather than a comprehensive validated measure of psychosocial impact.

#### 2.4.7. Data collection procedure and quality control

After written informed consent was obtained, participants underwent a clinical oral examination and independently completed the psychological scales without researcher guidance. Questionnaires were collected on-site and checked for completeness; questionnaires with >20% missing data on key items were considered invalid. Data were entered independently by 2 individuals using EpiData software and subjected to logical verification to support data accuracy.

### 2.5. Statistical analysis

Data were analyzed using IBM SPSS Statistics version 26.0 (IBM Corp.). Continuous variables were summarized as mean ± standard deviation and categorical variables as frequency (percentage). One-way analysis of variance with post hoc least significant difference comparisons and chi-square tests were used for between-group comparisons. Spearman’s correlation analysis was used to examine associations among the main variables. Multiple linear regression models were constructed with total social anxiety score and total self-esteem score as dependent variables, respectively, and malocclusion group (dummy coded), age, gender, monthly family income, and perceived social support as independent variables. Regression coefficients were interpreted as adjusted associations rather than causal effects. Exploratory interaction terms (subgroup variable × malocclusion group) were used to examine possible heterogeneity by gender, history of being teased, and concern about dental appearance. Because these subgroup analyses were exploratory and involved multiple comparisons, their results were considered hypothesis-generating and were not used to establish definitive subgroup-specific effects. Statistical significance was set at α = 0.05.

## 3. Results

### 3.1. Baseline characteristics of the study participants

A total of 190 adolescents were included in this study, with a mean age of (14.8 ± 1.9) years, including 92 males (48.4%). Based on the IOTN-DHC, participants were divided into a mild (n = 62), moderate (n = 71), and severe malocclusion group (n = 57). There were no significant differences in baseline characteristics such as age, gender, or family income among the 3 groups (*P* > .05), indicating comparability (Table 1).

**Table 1:** Sociodemographic, clinical, and psychological characteristics of study participants.

Characteristic	Total (n = 190)	Mild malocclusion group (n = 62)	Moderate malocclusion group (n = 71)	Severe malocclusion group (n = 57)	Statistic	*P*-value
Sociodemographic characteristics						
Age (yr), mean ± SD	14.8 ± 1.9	14.6 ± 2.0	15.0 ± 1.8	14.8 ± 1.9	*F* = 0.725	.486
Gender, n (%)					χ^2^ = 0.842	.656
Male	92 (48.4)	28 (45.2)	37 (52.1)	27 (47.4)		
Female	98 (51.6)	34 (54.8)	34 (47.9)	30 (52.6)		
Monthly family income, n (%)					χ^2^ = 4.562	.335
<5000 RMB	45 (23.7)	12 (19.4)	20 (28.2)	13 (22.8)		
5000–10,000 RMB	78 (41.1)	30 (48.4)	26 (36.6)	22 (38.6)		
>10,000 RMB	67 (35.3)	20 (32.3)	25 (35.2)	22 (38.6)		
Clinical characteristics						
Primary type of malocclusion, n (%)						
Angle class I	85 (44.7)	35 (56.5)	32 (45.1)	18 (31.6)		
Angle class II	68 (35.8)	18 (29.0)	28 (39.4)	22 (38.6)		
Angle class III	37 (19.5)	9 (14.5)	11 (15.5)	17 (29.8)		
History of prior orthodontic consultation, n (%)	78 (41.1)	18 (29.0)	30 (42.3)	30 (52.6)	χ^2^ = 7.215	.027
Psychosocial characteristics						
Perceived social support total score, mean ± SD	62.5 ± 9.8	64.1 ± 8.5	62.0 ± 10.1	61.5 ± 10.5	*F* = 1.234	.293
Concern about dental appearance (VAS 0–10), mean ± SD	5.8 ± 2.5	4.5 ± 2.0	5.9 ± 2.3	7.2 ± 2.4	*F* = 25.736	<.001
History of being teased about teeth, n (%)	65 (34.2)	12 (19.4)	23 (32.4)	30 (52.6)	χ^2^ = 15.873	<.001

SD = standard deviation, VAS = Visual Analogue Scale.

Analysis of clinical and psychosocial characteristics revealed that Angle Class III malocclusion had the highest proportion in the severe group (29.8%). The proportion of participants with a history of prior orthodontic consultation increased with malocclusion severity (mild group: 29.0% vs severe group: 52.6%, *P* = .027). Significant differences were observed in indicators reflecting subjective patient experiences. Concern about dental appearance (VAS score) increased from (4.5 ± 2.0) in the mild group to (7.2 ± 2.4) in the severe group (*P* < .001). The proportion of participants who had been teased about their teeth increased from 19.4% (mild group) to 52.6% (severe group; *P* < .001). No significant difference was found in perceived social support levels among the 3 groups (*P* = .293).

### 3.2. Comparison of main psychological variables between groups

Social anxiety and self-esteem scores were compared across the 3 malocclusion groups (Table 2). LSAS total, fear, and avoidance scores showed a stepwise increase across increasing malocclusion categories (all *P* < .001). Pairwise comparisons indicated that the severe group had significantly higher scores than the mild and moderate groups (*P* < .001), whereas the mild and moderate groups did not differ significantly. The proportion scoring above the LSAS screening threshold was 64.9% in the severe group, 29.6% in the moderate group, and 14.5% in the mild group (*P* < .001). RSES scores were lower with increasing malocclusion severity (*P* < .001), with the severe group showing significantly lower self-esteem scores than the other 2 groups. These cross-sectional comparisons describe associations and do not establish that malocclusion caused the psychological differences.

**Table 2:** Comparison of social anxiety and self-esteem scores between groups with different malocclusion severity levels (mean ± SD).

Variable	Mild malocclusion group (n = 62)	Moderate malocclusion group (n = 71)	Severe mamlocclusion group (n = 57)	*F*/χ^2^ value	*P*-value
LSAS-total score	28.4 ± 10.2	33.7 ± 12.5	48.6 ± 15.8	45.32	<.001
Fear subscale	13.8 ± 5.1	16.5 ± 6.3	23.9 ± 8.0	38.76	<.001
Avoidance subscale	14.6 ± 5.8	17.2 ± 6.9	24.7 ± 8.5	39.21	<.001
Clinical anxiety risk, n (%)	9 (14.5%)	21 (29.6%)	37 (64.9%)	χ^2^ = 32.15	<.001
RSES-total score	32.5 ± 5.1	30.8 ± 5.6	26.3 ± 6.2	24.18	<.001

LSAS = Liebowitz Social Anxiety Scale, RSES = Rosenberg Self-Esteem Scale, SD = standard deviation.

### 3.3. Correlation analysis between variables

To preliminarily explore the direction and strength of associations among the main study variables, Spearman’s correlation analysis was conducted. The results (Table 3) showed that malocclusion severity was significantly positively correlated with the total social anxiety score (*r*_s_ = 0.35, *P* < .001) and significantly negatively correlated with the total self-esteem score (*r*_s_ = −0.31, *P* < .001). A strong negative correlation was found between social anxiety and self-esteem (*r*_s_ = −0.52, *P* < .001).

**Table 3:** Spearman correlation matrix for malocclusion severity, social anxiety, and self-esteem.

Variable	1	2	3
1. Malocclusion sverity	1		
2. Social anxiety total score	0.35**	1	
3. Self-esteem total score	−0.31**	−0.52**	1

* indicates *P* < .01.

** indicates *P* < .001.

### 3.4. Adjusted associations between malocclusion severity and psychosocial outcomes

Multiple linear regression was performed to estimate adjusted associations between malocclusion severity and psychosocial outcomes (Table 4). Compared with the mild malocclusion group, the severe malocclusion group was associated with higher social anxiety (β = 0.52, *P* < .001) and lower self-esteem (β = −0.41, *P* < .001) after adjustment for measured covariates. Differences between the moderate and mild groups were not statistically significant. Higher perceived social support was independently associated with lower anxiety (β = −0.19, *P* = .002) and higher self-esteem (β = 0.32, *P* < .001). The models explained 46.8% of the variance in anxiety and 38.2% of the variance in self-esteem, respectively. These coefficients represent independent correlates within a cross-sectional model and should not be interpreted as causal effects.

**Table 4:** Multiple linear regression analysis of factors influencing social anxiety and self-esteem levels.

Predictors	Model 1: social anxiety total score				Model 2: self-esteem total score			
	B (95% CI)	β	*t* value	*P*-value	B (95% CI)	β	*t* value	*P*-value
Constant	45.12 (32.18, 58.06)	–	6.85	<.001	18.45 (12.33, 24.57)	–	5.94	<.001
Age	0.58 (−0.21, 1.37)	0.07	1.45	.149	−0.22 (−0.64, 0.20)	−0.06	−1.03	.303
Gender (Reference: male)								
Female	1.89 (−1.45, 5.23)	0.05	1.11	.268	−1.05 (−2.77, 0.67)	−0.07	−1.20	.232
Monthly family income (Reference: <5000 RMB)								
5000–10,000 RMB	−1.02 (−5.01, 2.97)	−0.03	−0.50	.616	0.89 (−0.99, 2.77)	0.06	0.93	.354
>10,000 RMB	−2.15 (−6.38, 2.08)	−0.05	−1.00	.319	1.34 (−0.66, 3.34)	0.08	1.32	.189
Perceived social support	−0.31 (−0.51, −0.11)	−0.19	−3.05	.002	0.21 (0.14, 0.28)	0.32	5.85	<.001
Malocclusion (Reference: mild group)								
Moderate group	4.05 (−0.10, 8.20)	0.11	1.93	.055	−1.52 (−3.38, 0.34)	−0.11	−1.61	.110
Severe group	18.24 (13.78, 22.70)	0.52	8.09	<.001	−5.68 (−7.66, −3.70)	−0.41	−5.66	<.001
Model summary	Adjusted *R*^2^ = 0.468, *F* = 21.35, *P* < .001				Adjusted *R*^2^ = 0.382, *F* = 15.42, *P* < .001			

CI = confidence interval.

### 3.5. Subgroup analysis of the impact of malocclusion on social anxiety

Exploratory subgroup analyses examined whether the association between severe malocclusion and social anxiety differed by gender, history of being teased, and concern about dental appearance (Table 5). The estimates suggested possible heterogeneity: the association was larger in females (β = 0.35) than in males (β = 0.24; *P* for interaction = .042), among adolescents who reported dental teasing (β = 0.45 vs 0.18; *P* for interaction < .001), and among those with high concern about dental appearance (β = 0.38 vs 0.20; *P* for interaction = .015). Because these analyses were exploratory and multiple interactions were tested, the findings should be interpreted as hypothesis-generating rather than definitive evidence of subgroup-specific effects.

**Table 5:** Comparison of the impact of malocclusion severity on total social anxiety score across different subgroups.

Subgroup (stratification variable)	Subgroup sample size	Severe vs mild malocclusion (reference)		*P*-value for interaction	Model adjusted *R*^2^
		β	*P*-value		
1. Gender				.042	
Male	92	0.24	0.002		0.218
Female	98	0.35	<0.001		0.327
2. History of being teased about teeth				<.001	
Yes	65	0.45	<0.001		0.401
No	125	0.18	0.023		0.198
3. Concern about dental appearance				.015	
High concern group (≥7 points)	68	0.38	<0.001		0.362
Low concern group (<7 points)	122	0.20	0.010		0.175

### 3.6. Exploratory subgroup analysis of the association with self-esteem

Exploratory subgroup analysis suggested that the association between severe malocclusion and lower self-esteem may have been stronger among adolescents who reported dental teasing (β = −0.37 vs −0.15; *P* for interaction = .008) and those with high concern about dental appearance (β = −0.34 vs −0.16; *P* for interaction = .009; Table 6). In gender-stratified analyses, the estimate was larger in females (β = −0.29 vs −0.21), but the interaction was not statistically significant (*P* = .112). These patterns should be interpreted cautiously because the subgroup analyses were exploratory and were not designed to demonstrate causal mechanisms or definitive effect modification.

**Table 6:** Comparison of the impact of malocclusion severity on total self-esteem score across different subgroups.

Subgroup (stratification variable)	Subgroup sample size	Severe vs mild malocclusion (reference)		*P*-value for interaction	Model adjusted *R*^2^
		β	*P*-value		
1. Gender				.112	
Male	92	−0.21	0.008		0.185
Female	98	−0.29	<0.001		0.268
2. History of being teased about teeth				.008	
Yes	65	−0.37	<0.001		0.335
No	125	−0.15	0.045		0.162
3. Concern about dental appearance				.009	
High concern group (≥7 points)	68	−0.34	<0.001		0.312
Low concern group (<7 points)	122	−0.16	0.032		0.149

## 4. Discussion

In this cross-sectional study of 190 adolescents, greater malocclusion severity was associated with higher social anxiety and lower self-esteem. These associations persisted after adjustment for measured demographic factors and perceived social support. The findings are broadly consistent with previous studies reporting psychosocial differences according to malocclusion severity.^[13-15]^ However, the present design does not establish that malocclusion causes social anxiety or low self-esteem; severe malocclusion should therefore be described as an independent correlate or associated factor rather than a psychosocial risk factor.

No statistically significant differences in social anxiety or self-esteem were observed between the mild and moderate groups, whereas the severe group differed from both. This pattern is compatible with a possible threshold-like association, but it should not be interpreted as evidence of a biological or psychological threshold. The 3-category grouping combined IOTN-DHC Grades 1 to 2 and Grades 4 to 5, which may have obscured clinically meaningful differences between adjacent grades. In addition, IOTN-DHC primarily quantifies orthodontic treatment need based on dental health rather than psychosocial or aesthetic impact.^[16]^ Future studies should retain the full 5-grade IOTN-DHC distribution where sample size permits and consider complementary measures such as the IOTN Aesthetic Component and validated psychosocial or oral-health-related quality-of-life instruments. Such approaches would allow a more granular evaluation of whether psychological outcomes track clinical treatment need, perceived aesthetics, or both.

From a clinical perspective, this implies that orthodontists, when assessing treatment need, should consider not only functional factors and objective malocclusion indices but also the psychological state of patients with severe malocclusion, integrating this as an important component of the comprehensive treatment plan. The high proportion (64.9%) of clinical anxiety risk in the severe malocclusion group in this study further underscores the necessity of psychological screening for this population.^[17]^

The exploratory analyses also identified hypothesis-generating patterns involving dental appearance concern and teasing. Malocclusion severity was associated with these experiences, and the adjusted associations with anxiety and self-esteem appeared stronger in some adolescents who reported high appearance concern or a history of teasing. These data are consistent with, but do not demonstrate, a possible pathway in which objective dentofacial characteristics interact with subjective appraisal and social experiences.^[18]^ Alternative explanations are also plausible. For example, adolescents with higher social anxiety or lower self-esteem may perceive or report their dental appearance more negatively, may recall teasing differently, or may be more likely to seek orthodontic care. Thus, reverse causation and selection processes cannot be excluded, and mediation or mechanistic language is not justified without longitudinal temporal data. Future longitudinal studies could formally test whether appearance concern or teasing precedes, mediates, or modifies changes in psychological outcomes.^[19]^ Higher perceived social support was independently associated with lower social anxiety and higher self-esteem. This finding is consistent with the possibility that social support may be relevant to psychological resilience,^[20]^ but the cross-sectional data cannot establish a protective causal effect. Clinically, assessing available family and peer support may help identify adolescents who could benefit from additional psychosocial evaluation, while intervention studies are needed to determine whether strengthening social support improves outcomes.

Regarding gender, exploratory interaction analysis suggested a stronger association between severe malocclusion and social anxiety in females, whereas the interaction for self-esteem was not statistically significant. Prior research has described gender differences in appearance-related anxiety,^[21]^ but the present subgroup results should not be treated as definitive because of the limited sample size within strata and the multiple interaction tests. Replication in larger, prospectively designed studies is needed before gender-specific mechanisms or clinical recommendations can be inferred.

Several limitations warrant emphasis. First, the cross-sectional design precludes causal inference and does not establish temporality. Although malocclusion was clinically assessed, reverse causation or bidirectional processes may influence subjective appearance concern, recall/reporting of teasing, psychological symptoms, and treatment-seeking behavior. Longitudinal studies with repeated psychological assessments before and during orthodontic care are required to clarify directionality. Second, participants were recruited from a single hospital orthodontic/outpatient setting using a convenience sample, which may introduce selection bias and limit generalizability to community adolescents or those not seeking care. Third, complete recruitment-flow information (numbers screened, excluded with reasons, and refusals) should be reported from the original screening log to permit assessment of selection processes. Fourth, psychological variables were based on self-report instruments; the LSAS threshold represents screening risk rather than a psychiatric diagnosis, and the single-item Visual Analogue Scale for dental appearance concern is not a comprehensive psychosocial measure. Fifth, combining IOTN-DHC Grades 1 to 2 and Grades 4 to 5 improved clinical interpretability of the 3-group analysis but reduced severity granularity and may have masked differences between Grade 1 versus 2 and Grade 4 versus 5. IOTN-DHC also reflects treatment need rather than psychosocial impact. Future studies should consider grade-specific analyses and complementary aesthetic/psychosocial measures. Sixth, the subgroup interaction analyses were exploratory, involved multiple comparisons, and may be unstable in modest strata; they require independent replication. Finally, unmeasured confounding remains possible despite multivariable adjustment. Multi-center longitudinal studies with community sampling and repeated measures are needed to test temporal and potentially mechanistic hypotheses.

Despite these limitations, the findings have potential clinical relevance. Adolescents with severe malocclusion in this clinical sample more often reported elevated social anxiety and lower self-esteem, particularly in some exploratory subgroups characterized by teasing or high appearance concern. Orthodontic assessment may therefore provide an opportunity to ask about psychosocial distress and, when indicated, consider referral for appropriate psychological evaluation or support. These findings do not show that orthodontic treatment itself will improve mental health, and intervention or longitudinal studies are needed to determine whether changes in malocclusion, appearance concern, or social experiences are followed by changes in psychological outcomes.

## Author contributions

**Conceptualization:** Wanjun Zhang, Yongping Ma, Yanming Guo, Qian Yang, Zhen Da.

**Data curation:** Wanjun Zhang, Yongping Ma, Yanming Guo, Qian Yang, Zhen Da.

**Formal analysis:** Wanjun Zhang, Yongping Ma, Yanming Guo, Qian Yang, Zhen Da.

**Funding acquisition:** Zhen Da.

**Investigation:** Zhen Da.

**Writing – original draft:** Zhen Da.

**Writing – review & editing:** Zhen Da.
